# Immune system development during early childhood in tropical Latin America: Evidence for the age-dependent down regulation of the innate immune response

**DOI:** 10.1016/j.clim.2010.12.011

**Published:** 2011-03

**Authors:** Rommy Teran, Edward Mitre, Maritza Vaca, Silvia Erazo, Gisela Oviedo, Marc P. Hübner, Martha E. Chico, Joseph J. Mattapallil, Quentin Bickle, Laura C. Rodrigues, Philip J. Cooper

**Affiliations:** a Laboratorio de Investigaciones FEPIS, Quininde, Esmeraldas Province, Ecuador; b Colegio de Ciencias de la Salud, Universidad San Francisco de Quito, Av. Interoceanica Km 12.5 y Av. Florencia, Cumbaya, Quito, Ecuador; c Department of Microbiology and Immunology, Uniformed Services University of the Health Sciences, 4301 Jones Bridge Rd, Bethesda, MD 20814, USA; d Institute for Medical Microbiology, University Hospital Bonn, 53105 Bonn, Germany; e Immunology Unit, London School of Hygiene and Tropical Medicine, Keppel Street, London WC1E 7HT, UK; f Department of Epidemiology, London School of Hygiene and Tropical Medicine, Keppel Street, London WC1E 7HT, UK; g Molecular and Biochemical Parasitology, Liverpool School of Tropical Medicine, Liverpool L3 5QA, UK

**Keywords:** SEB, *Staphylococcus* enterotoxin B, LA, Latin America, T Regs, regulatory T cells, Innate immunity, Adaptive immunity, Childhood, Tropics

## Abstract

The immune response that develops in early childhood underlies the development of inflammatory diseases such as asthma and there are few data from tropical Latin America (LA). This study investigated the effects of age on the development of immunity during the first 5 years of life by comparing innate and adaptive immune responses in Ecuadorian children aged 6–9 months, 22–26 months, and 48–60 months. Percentages of naïve CD4+ T cells declined with age while those of memory CD4^+^ and CD8^+^ T cells increased indicating active development of the immune system throughout the first five years. Young infants had greater innate immune responses to TLR agonists compared to older children while regulatory responses including SEB-induced IL-10 and percentages of FoxP3^+^ T-regulatory cells decreased with age. Enhanced innate immunity in early life may be important for host defense against pathogens but may increase the risk of immunopathology.

## 1 Introduction

The prevalence of inflammatory allergic diseases has increased in industrialized countries over recent decades [1], although such diseases remain relatively rare in rural environments, particularly in the Tropics. Environmental exposures are important determinants of allergic disease risk and those occurring during early life are considered to be most relevant [2]. Microbial exposures may protect against the development of allergic diseases [3,4] and mediate their effects by driving an early maturation of the innate and adaptive immune responses [5,6] that are immature at the time of birth.

Studies conducted in industrialized countries have shown that neonates have an impaired capacity to produce and respond to T_H_1 cytokines [7]. Development of T_H_1 immunity has been attributed to microbial stimulation of innate immunity. After birth, the progressive up-regulation of T_H_1 function towards adult-levels may occur through stimulation of innate immunity by microbes in the environment [6]. Delayed T_H_1 maturation associated with a failure to down-regulate T_H_2 responses may contribute to the impaired regulation that underlies the development of allergic diseases such as asthma [6].

There are very limited data on the determinants of immune responses during early childhood in the rural Tropics. Such factors may provide clues to why the risk of allergic disease appears to be low in populations living in the rural Tropics. In the present study, we investigated age-dependent changes in early immune responses and the role of early environment in shaping these by measuring cytokine responses to T cell and innate immune stimuli and phenotypic T cell markers in comparable groups of children aged between 6 months and 5 years living in rural and urban environments in a tropical region of Latin America.

## 2 Materials and methods

### 2.1 Study design and population

Children in this cross-sectional study were recruited at two sites: 8 rural communities in the Districts of Eloy Alfaro and San Lorenzo in Esmeraldas Province and from 16 day care centers in the city of Esmeraldas (the principal city in the Province of Esmeraldas with a population of approximately 250,000). The rural communities were approximately 70–100 km distance (“as the crow flies”) from the city of Esmeraldas. All rural communities were predominantly Afro-Ecuadorian and urban children were selected from districts with high concentrations of Afro-Ecuadorian migrants from the rural study Districts. Only Afro-Ecuadorian children living in the study areas were eligible for inclusion. Children were recruited into 3 age groups to represent different stages of immune maturation: 6–9 months (early); 22–26 months (intermediate); 48–60 months (advanced). The present study was linked to a cross-sectional analysis of school-age children to investigate risk factors for asthma and atopy in urban and rural populations, and has been described in detail elsewhere [8]. Detailed information on demographic and lifestyle factors and allergic symptoms was obtained by parental questionnaire. Data on wheeze within the previous 12 months was obtained by parental questionnaire using the ISAAC phase II protocol [8]. Informed written parental consent was obtained for all children. Data collection was performed between March and August 2007. The protocol was approved by the ethics committees of the Universidad San Francisco de Quito and Hospital Pedro Vicente Maldonado.

### 2.2 Collection and analysis of stool and blood samples

Single stool samples were collected and examined for the presence of helminth eggs and larvae using the modified Kato–Katz and the formol-ethyl acetate-concentration methods [9]. Blood samples (3 ml) were collected into plastic tubes (BD Vaccutainer) containing sodium heparin as an anticoagulant, and were analyzed within 5 hours of collection. Differential and white cell counts were calculated using standard laboratory procedures.

### 2.3 Skin-prick testing for allergens

Skin-prick testing was performed by the study physician (MV). Allergens were pricked onto skin of the forearm or back and reactions were measured after 15 minutes. Allergen extracts used were: house dust-mite (*Dermatophagoides pteronyssinus*; Greer Laboratories), grass/pollen mix (Greer Laboratories), American cockroach (*Periplaneta americana*; Greer Laboratories), fungi mix (Greer Laboratories), *Alternaria tenuis* (Greer Laboratories), cat (Greer Laboratories), dog (Greer Laboratories), *Blomia tropicalis* (Laboratorios Leti), and *Chortoglyphus arcuatus* (Laboratorios Leti). Histamine (ALK-Abello) and saline solution (ALK-Abello) were used as positive and negative controls, respectively. A reaction was considered positive if the mean wheal diameter was ≥ 3 mm greater than the negative control.

### 2.4 Whole blood culture

Whole blood was cultured 1:4 diluted in RPMI 1640 GlutaMAX™-I (Invitrogen) containing 80 mg/ml gentamicin (GIBCO™) and 1% HEPES (Biowhittaker). Diluted whole blood (0.5 ml) was cultured alone or with 2 μg/ml *Staphylococcus* enterotoxin B (SEB; Sigma-Aldrich) or TLR agonists (Invivogen), Pam3CSK4-TLR1/2 (100 ng/ml), Poly (I:C)-TLR3 (25 μg/ml), *E. coli* K12 LPS-TLR4 (100 ng/ml), *S. Typhimurium* Flagellin-TLR5 (100 ng/ml), FLS1-TLR6/2 (200 ng/ml), imiquimod-TLR7 (1 μg/ml), ssRNA40-TLR8 (100 ng/ml), and ODN 2006 (Type B)-TLR9 (1uM). Cultures were incubated in a humidified atmosphere of 5% CO_2_ at 37 °C. Supernatants were collected at 24 hours (TLR agonists) or 5 days (SEB) and stored at −70 °C until analysis.

### 2.5 Cytokine assays

Cytokines and chemokines from cell culture supernatants were quantified according to manufacturer's instructions by sandwich ELISA using antibody pairs: IL-13, IL-5, IL-8 and TNF-α (BD Biosciences Pharmingen) and IL-10, IL-6 and IFN-γ (R&D DuoSet). The absorbance was measured using a Molecular Devices Vmax plate reader and SOFTmax Pro 4.0 software. The detection limits for the assays were: for IFN-γ (63 pg/ml), IL-13 (234 pg/ml), IL-5 (63 pg/ml), IL-10 (78 pg/ml), IL-6 (2.3 pg/ml), IL-8 (1.6 pg/ml), and TNF-α (39 pg/ml). Values below this limit were assigned the half of the detection limit.

### 2.6 Flow cytometric analyses

**Figure 1:**
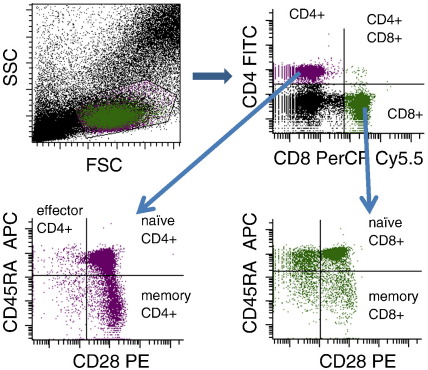
Flow cytometry gating strategy for CD4^+^ and CD8^+^ T-cell subsets in whole blood samples. Lymphocytes were initially gated by forward and side scatter properties (upper left panel). CD4^+^ T-cells and CD8^+^ T-cells (upper right panel) were then gated and assessed for surface expression of CD45RA and CD28.

**Figure 2:**
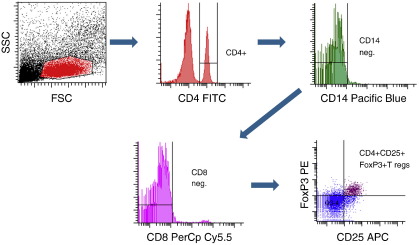
Flow cytometry gating strategy for CD4^+^CD25^+^FoxP3^+^ T-regulatory cells.

Whole-blood was resuspended in 10% FACS lysing solution (BD Biosciences) and incubated for 10 min. Cells were then washed once in cold PBS, centrifuged at 400gr 10 min, and fixed with 4% paraformaldehyde (Fluka) for 5 min, and cryopreserved in PBS/10%DMSO. Cells were stored at −70 °C and shipped to USUHS on dry ice for analysis. After thawing, cells were washed once with PBS/1%BSA (Sigma), followed by blocking with PBS/1% BSA for 1 hour at room temperature. To identify regulatory T cells (CD4^+^FoxP3^+^CD25^+^CD14^-^CD8^-^), cells were permeabilized for 30 minutes at room temperature (permeabilization buffer eBioscience) and stained for five-color-flow cytometry with mouse-anti-human CD4 FITC (BD Pharmingen), mouse-anti-human FoxP3 PE (BD Pharmingen), mouse-anti-human CD25 allophycocyanin APC (BD Pharmingen), mouse-anti-human CD14 Pacific blue (BD Pharmingen) and mouse-anti-human CD8 PerCP Cy5.5 (eBioscience). Detection of naïve (CD45RA^+^CD28^+^), memory (CD45RA^−^CD28^+^) and CD45RA^+^CD28^-^ (a population of cells that may represent effector cells [10,11]) CD4^+^ and CD8^+^ cell populations was performed using four-color-flow cytometry with mouse-anti-human CD4 FITC (BD Pharmingen), mouse-anti-human CD8 PerCP Cy5.5 (eBioscience), mouse-anti-human CD45RA allophycocyanin (eBioscience) and mouse-anti-human CD28 PE (eBioscience). Flow cytometry was performed using the BD LSRII system and subsequently analyzed with FACSDiVa 6.1 software (BD Biosciences). All flow cytometry antibodies were individually titrated and, prior to each experiment, compensation was conducted with BD CompBeads (BD Biosciences) bound to the flow cytometry antibodies used in that experiment. Gating strategies for percentages of CD4^+^ and CD8^+^ T cell subsets and CD4^+^CD25^+^FoxP3^+^ regulatory T cells are shown in Figures 1 and 2, respectively. A total of 150,000 events were acquired for the calculation of percentages of memory, naïve, and CD45RA^+^CD28^-^ T cell populations and 200,000 for T reg percentages. The percentages of T cell subsets represent the proportion of CD4^+^ (or CD8^+^) T cells that express the phenotypic markers for naïve, memory, CD45RA^+^CD28^-^, and regulatory cells. Absolute cell counts for each T cell population were estimated from peripheral blood lymphocyte counts.

### 2.7 Statistical analysis

Blood samples in both urban and rural populations were collected and analyzed (blood culture and ELISA) independent of age. Cytokine and chemokine concentrations were analyzed as continuous or binary (responder versus non-responder) variables defined by the lower limits of detection of the assays. Differences between groups (defined by age or urban-rural residence) for continuous variables were analyzed using the Mann-Whitney test for 2 groups, the Kruskal–Wallis test for more than 2 groups and for binary variables using the Chi-square test. Associations between continuous variables were assessed by calculation of Spearman's rank correlation coefficients. To investigate the environmental exposures that may mediate the effects of residence on the immune parameters, we used linear regression to evaluate the associations between log_e_-transformed immune parameters that showed significant effects for residence and potential environmental exposures that differed between the urban and rural populations shown in Table 1. For these analyses, in the case of T cell populations, we used percentages rather than absolute cell counts because of greater potential for measurement error for the latter. Results of linear regression were back-transformed to provide fold-change in geometric means. Residence was excluded as a variable from multivariate models. Because of multiple comparisons, only *P*-values lower than 0.01 were considered to be statistically significant. Statistical analyses were performed with STATA 10 software.

## 3 Results

### 3.1 Study population

Baseline characteristics of the children in urban and rural study areas are shown in Table 1. All children were Afro-Ecuadorian. Median age, maternal educational level, household material goods, and median monthly income were similar for urban and rural children. A higher proportion of girls than boys were recruited in the urban compared to rural samples. There were no differences between urban and rural children with respect to socioeconomic factors (median monthly income and household electrical appliances), maternal educational level, breast-feeding ever (rural 98.2% vs. urban 99.2%, *P* = 0.48) or duration, crowding, birth order, or presence of BCG (Bacille Calmette-Guérin) scars (rural 96.4% vs. urban 95.2%, *P* = 0.67). There were differences between the two groups with respect to type of bathroom, sources of drinking water, and household construction materials. The majority of rural (83%) and urban (91%) children lived in their place of birth. Hematologic and nutritional characteristics, allergen skin test reactivity, frequency of recent wheeze, and geohelminth infections stratified by age and residence group are shown in Table 2. Nutritional and hematologic characteristics were similar for children living in urban and rural environments for each of the 3 age groupings except: 1) eosinophil counts were greater in rural compared to urban children aged 22–26 months (*P* = 0.02); and 2) neutrophil counts were greater in urban versus rural children aged 48–60 months (*P* = 0.004). The majority of rural children aged 22–26 months were infected with geohelminths (62.2%) while infections were acquired later in urban children. Positive allergen skin prick tests, mainly to house dust mite, reached adult levels (~ 10% in both urban and rural populations, data not shown) at 22–26 months in urban compared to 48–60 months in rural children. High proportions of both urban and rural children had wheeze over the previous 12 months at or before 2 years of age that were likely to be associated with viral respiratory tract infections.

### 3.2 Effects of age on immune parameters

To investigate innate immune responses, we analyzed the release of IL-6, IL-8, IL-10, and TNF-α by blood stimulated with agonists for TLR 1–8. Responses to TLR 7 and 8 were negligible (data not shown). Significant age-dependent falls in TLR responses were observed for the chemokine (IL-8) and cytokines measured as follows (Table 3): IL-6 –TLR4 (*P* = 0.005), TLR6 (*P* = 0.0004); IL-8–TLR1/2 (*P* = 0.0003), TLR4 (*P* = 0.001), TLR5 (*P* = 0.0001), TLR6 (*P* = 0.0001), and TLR9 (*P* = 0.0001); IL-10–TLR1/2 (*P* = 0.001), TLR4 (*P* = 0.004), TLR5 (*P* = 0.009), TLR6 (*P* = 0.0001), and TLR9 (*P* = 0.006); TNF-α–TLR6 (*P* = 0.0002).

T cell cytokine responses were measured using SEB, a polyclonal activator of T cells (Table 4). An age-dependent decline in SEB-induced IL-10 (*P* = 0.0006) was observed. There was evidence also for an increase in IL-5 (*P* = 0.0001) with age, most of this effect being observed between the 6–9 and 22–26 month groups. Levels of IFN-γ and IL-13 were similar across the 3 age groups.

The percentages of CD4^+^ T-cells that were naive (CD45RA^+^CD28^+^) decreased across age groups (*P* = 0.0001) while those displaying a memory phenotype (CD45RA^−^CD28^+^) increased with age (*P* = 0.0001) (Table 5). Age-dependent increases in numbers of memory CD4^+^ T cells were observed also (*P* = 0.002). The percentages of CD4^+^ and CD8^+^ T-cells with the phenotype CD45RA^+^CD28^-^, that may represent effector cells [10,11], were similar in all age groups, although when expressed as cell counts there was evidence for significant age-dependent declines for CD4^+^ (*P* = 0.001) and CD8^+^ cells of this phenotype (*P* = 0.001). While the percentage of CD8^+^ T-cells that exhibited a naive phenotype did not change significantly with age, the percentage and numbers of memory CD8^+^ T cells increased significantly (percentage, *P* = 0.0001; cell counts, *P* = 0.0001). The percentage and numbers of circulating CD4^+^ T-cells that were regulatory T cells (CD4^+^CD25^+^FoxP3^+^) decreased with age (percentage, *P* < 0.0001; cell counts, *P* = 0.001) (Table 5).

### 3.3 Effects of environment on immune parameters

The effect of early environment (urban vs. rural) on immune responses was evaluated in the earliest age grouping (6–9 months) because the immune response is likely to be most plastic with respect to the modifying effects of environment in the youngest compared to older age groupings. IFN-γ (*P* = 0.001) and IL-10 (*P* = 0.006) production were significantly elevated in urban compared to rural infants at this age, but there were no differences in production of the Th2 cytokines, IL-5 and IL-13 (Table 4).

There was evidence of increased percentages of memory CD8^+^ T cells in rural compared to urban children (*P* = 0.004), but not for any of the other phenotypic markers measured (Table 5). No significant effects of residence were observed when T cell sub-groups were expressed as absolute counts.

Generalized reduced IL-8 responses but not TNF-α, IL-6, and IL-10 responses were seen in urban versus rural infants for whole blood stimulated with TLR1/2 (*P* = 0.001), TLR3 (*P* = 0.0001), TLR 4 (*P* = 0.005), and TLR 5 (*P* = 0.002).

Comparisons between urban and rural children, respectively, for the other two age groupings showed: 1) in children aged 22–26 months, lower production of IL-10 to TLR9 (*P* = 0.002), percentages (*P* = 0.004) and counts (*P* = 0.002) of regulatory FoxP3^+^ T cells, and counts of memory CD8^+^ (*P* = 0.01) and CD8^+^CD45^+^CD28^-^ T cells (*P* = 0.002), but increased SEB-induced IL-10 (*P* = 0.0002); 2) in children aged 48–60 months, lower production of IL-6 to TLR3 (*P* = 0.001), percentages (*P* < 0.0001) and counts (*P* < 0.0001) of CD4^+^CD45RA^+^CD28^+^ T cells, and counts of regulatory FoxP3^+^ T cells (*P* = 0.006), CD8^+^ T cells (*P* < 0.0001), and CD8^+^CD45RA^+^CD28^-^ T cells (*P* = 0.003), but increased production of IL-6 (*P* = 0.009) and TNF-α to TLR5 (*P* = 0.002), counts of memory CD4^+^ T cells (*P* = 0.004), and production of IL-10 by SEB-stimulated whole blood (*P* < 0.0001).

### 3.4 Effects of age, environment, and individual environmental exposures on immune parameters

**Figure 3:**
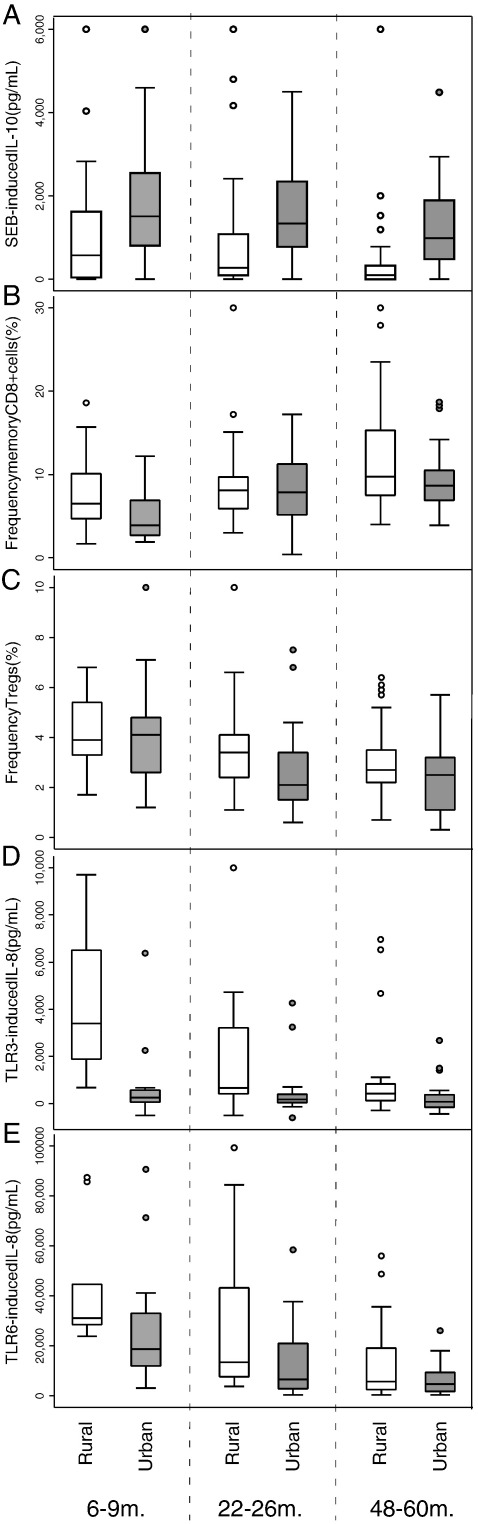
Immune parameters that showed independent effects for age and residence. Shown are residence effects (rural vs. urban) by age group (6–9 months, 22–26 months, and 48–60 months). Parameters are: A. SEB-induced IL-10; B. CD8^+^ memory T cells; C. Percentages of Fox P3^+^ T regs; D. TLR3-induced IL-8; E. TLR6-induced IL-8.

Linear regression models of the immune parameters showed significant independent effects of age and residence for SEB-induced IL-10 (age, *P* = 0.001; residence, *P* < 0.001), the percentages of FoxP3^+^ T regs (age, *P* < 0.001; residence, *P* < 0.001) and memory CD8^+^ T cells (age, *P* < 0.001, residence, *P* < 0.001), and IL-8 production by blood stimulated with TLR3 (age, *P* = 0.004; residence, *P* < 0.001) and TLR6 (age, *P* < 0.001; residence, *P* = 0.001). Differences in levels of these 5 immune parameters by age and residence are shown in Figure 3. Specifically, increasing age was associated with decreasing IL-10 production in response to T-cell activation, decreasing percentages of FoxP3^+^ T regs, decreasing IL-8 release in response to TLR3 and TLR6 ligands, and increased numbers of memory CD8^+^ T-cells. Significant effects of age but not residence were seen for SEB-induced IL-5 (*P* < 0.001), percentages of naïve (*P* < 0.001) and memory (*P* < 0.001) CD4^+^ T cells, IL-8 production by blood stimulated with TLR1/2 (*P* = 0.001), TLR4 (*P* = 0.002), TLR5 (*P* < 0.001), and TLR9 (*P* < 0.001); and IL-10 production after stimulation with TLR6 (*P* < 0.001) and TLR9 (*P* = 0.006). Significant effects of residence but not age were not seen for any of the immune parameters.

Immune development as measured by increasing percentages of memory and decreasing percentages of naïve T cells was compared between urban and rural children for all age groups. Percentages of memory T cells were similar in both environments at all 3 age groups, but percentages of naïve CD4^+^ T cells were greater in urban compared to rural children aged 48–60 months (*P* = 0.0001).

We explored which specific environmental exposures might mediate the effects of residence on the 5 immune parameters that showed independent effects for residence (SEB-induced IL-10, percentages of FoxP3^+^ T regs and CD8+ memory T cells, and TLR3- and TLR6-induced IL-8 (Fig. 3)). Piped water was used as the drinking water exposure because there was no heterogeneity of exposure for river and potable water between urban and rural groups. We observed strong effects of residence (urban vs. rural) on all these parameters after controlling for age: SEB-induced IL-10 (Table 6, fold change 8.56, 95% CI 4.61–15.91, *P* < 0.001), percentages of regulatory (FC 0.82, 95% CI 0.74–0.91, *P* < 0.001) and memory (FC 0.79, 95% CI 0.68–0.90, *P* < 0.001) T cells, and TLR3 (FC 0.18, 95% CI 0.10–0.33, *P* < 0.001) and TLR6-induced production of IL-8 (FC 0.52, 95% CI 0.35–0.77, *P* = 0.001). Multivariate models that excluded residence showed that SEB-induced IL-10 (Table 6) increased with greater levels of household crowding (FC 1.26, 95% CI 1.12–1.62, *P* = 0.002). There were no significant associations observed between the other immune parameters and individual environmental exposures in multivariate analysis.

## 4 Discussion

In the present study, we investigated the effects of age and environment on several innate and T cell immune parameters in children living in a tropical region of Ecuador. The findings show that immune development continues to occur until at least 5 years of age as indicated by the increasing percentages of memory CD4^+^ and CD8^+^ T-cells and decreasing percentages of naïve CD4^+^ T cells. Further, this maturation of the immune system was associated with a strong age-dependent down-regulation of pro-inflammatory innate immune responses during the first 5 years of life, an observation that was linked to a decline in the percentages of regulatory T cells (CD4^+^CD25^+^FoxP3^+^) and IL-10 production by SEB-stimulated peripheral blood leukocytes.

We recruited urban infants and children of the same ethnicity (Afro-Ecuadorian) of parents that had migrated from the same rural Districts as used for the rural sampling. ‘Matching’ by ethnicity likely will have reduced recall biases from parental questionnaires and confounding by factors associated with ethnicity. Because we conducted our stimulations using *in vitro* stimulation of whole blood samples, the results we observed are likely a fairly accurate representation of what occurs during viremias or bacteremias of humans. However, because the individual cell types that make up the population of circulating blood cells likely differ as we age, we are not able to discern from our results whether changes in response to TLR agonists are due to intrinsic decreases in responsiveness to these molecules or whether this phenomenon occurs due to a changes in subsets of circulating blood cells. Another potential limitation was that we did not collect data earlier in infancy. Such data would have provided an important baseline with which to interpret age-dependent trends. The observation of significant levels of IFN-γ by 6–9 months and no age-dependent up-regulation in production of this cytokine, often referred to as a marker of immune maturation [7], may indicate that IFN- γ responses had matured markedly by 6–9 months. However, the finding that memory T-cells progressively increased with increasing age, along with a corresponding decrease in percentages of naïve T-cells, demonstrates that substantial immune system development continues through the first 5 years of life and occurred at similar rates in urban and rural populations.

To examine the effects of environment on immune responses we selected two distinct environments that we believed would differ fundamentally in terms of infectious and microbial exposures. We expected infectious exposures to be much greater in the rural environment because of poorer access to sanitation and clean drinking water compared to urban households (Table 1). Similarly we expected more intense environmental exposures to microbes (e.g. endotoxin) in the rural populations. Such exposures are considered to provide important signals for immune development in early life [12]. In fact, environment appeared had only modest effects on the immune parameters we measured. It is possible that many of those environmental exposures that influence immune maturation occurred with a similar intensity and at similar ages in both populations. Matching by ethnicity may have made our urban and rural populations more similar with respect to important environmental exposures - recent rural migrants to the city of Esmeraldas tend to settle at the periphery of the city and bring with them “ruralised” lifestyles [13] so several important environmental exposures may be shared by our urban and rural study populations.

The potential effects of environment on immunity were measured in infants aged 6 to 9 months, a time when we expected the immune system to be most plastic and subject to environmental influences. The effects observed by comparing data from urban with rural infants did show several interesting findings: 1) a stronger down-regulation of IL-10 responses but a delay in maximal SEB-induced IFN-γ responses in rural compared to urban infants; 2) a more rapid increase in memory CD8^+^ T cells in rural compared to urban infants; and 3) a more rapid down-regulation of IL-8 responses to TLR 3 and TLR6 in urban compared to rural children - similar but non-significant trends were observed for other TLR agonists. Few differences were observed for comparisons of urban and rural children for the older age groups. We observed only one significant association between a specific environmental exposure and immune parameter in multivariate analysis: an increase in SEB-induced IL-10 production with increasing levels of crowding. A study of children aged 4–11 years in urban Brazil showed an elevated frequency of IL-10 production (the spontaneous accumulation of IL-10 after 5 days of culture used as a marker for homeostatic immune regulation) by peripheral blood leukocytes (PBLs) from children without access to drinking water and sanitation in early life compared to children from more hygienic households [14]. In the present study we did not observe an effect of bathroom or water source on SEB-induced IL-10 after controlling for other covariates. Possible explanations are that the two parameters measure different regulatory effects or the fact that there is no municipal sewage drainage system or infrastructure for the provision of potable water that can be considered to be truly clean in urban Esmeraldas and that would be comparable to the city of Salvador in Brazil. No significant associations were observed between the immune parameters measured and geohelminth infection. Our data, therefore, provides only limited support that the different environmental exposures evaluated in this study have specific effects on different immune parameters. It is important to note, however, that the present study had limited power to detect effects for individual environmental exposures—larger and appropriately powered studies will be required to fully explore interactions between individual environmental exposures and immune parameters.

Previous studies have documented that the capacity of peripheral blood leukocytes to secrete a wide spectrum of inflammatory cytokines (e.g. IL-6, IL-10, TNF-α, and IFN- γ) is downregulated after birth [15]. LPS-induced production of IL-6 [15,16] and IL-8 [16] is elevated in neonates compared to young children [15] or adults [16], indicating an attenuation of inflammatory cytokine production postnatally [15]. In fact, neonatal mice appear to be highly susceptible to the pro-inflammatory effects of TLR agonists such as LPS and poly(I:C), a deficit that is related to the low numbers of T cells in neonates, making neonates susceptible to uncontrolled pro-inflammatory innate responses [17]. Our data showing an age-dependent decline in PBL responses to TLR agonists are consistent with these observations.

What then may be the consequences of enhanced innate immune responsiveness in early life? The ability of the innate immune system to respond to microbes may be critical in early life because of the immaturity of the adaptive immune response. However, an exaggerated innate immune response may contribute to the immunopathology of infection through excessive inflammatory cell recruitment and activation. For example, severe infections with respiratory syncytial virus in children have been linked to a higher capacity for LPS-induced production of IL-6 and IL-8 at birth [18]. In the present study, declines in IL-8 responses were strongly associated with percentages of FoxP3^+^ T regulatory cells that declined in parallel. The role of FoxP3^+^ T regs in modulating innate immunity is unclear - FoxP3^+^ T regs are predominantly of thymic origin [19] and can be considered ‘natural’ rather than ‘induced’. There is evidence that FoxP3^+^ T regs can directly suppress the activation of innate immune cells [20–22] although the ligation of TLRs of innate immune cells can also modulate the suppressive activity of FoxP3^+^ T regs [23]. IL-8 is a neutrophil activator and chemoattractant and, through its effects on neutrophils, likely provides a first line of defense against bacterial pathogens that are common causes of morbidity and mortality in early life. However, excessive neutrophil activation may carry severe immunopathological consequences, and it is likely that mechanisms exist that function to down-regulate such responses (e.g. FoxP3^+^ T regs) until the immune response matures sufficiently to respond to such infectious challenges in a more targeted and regulated manner.

The age-dependent decline in regulatory function observed in the present study (SEB-induced IL-10 and percentages of FoxP3^+^ T regs) was unexpected—we expected to observe age-dependent increases in both parameters. However, high levels of IL-10 production may have an important role early life in regulating the inflammatory response to infections. Greater mitogen-induced production of IL-I0 has been associated with a low risk of severe respiratory infections [24] and deficient IL-10 was associated with greater viral load following *in vivo* challenge with rhinovirus [25]. The source of IL-10 in SEB-stimulated peripheral blood is most likely to be T cells including populations of T regs although other cellular sources of IL-10 such as monocytes and B cells cannot be excluded. A study of Swedish infants showed that the percentages of CD4^+^CD25^+^FoxP3^+^ T cells increase after birth reaching a peak during the first month of life, but thereafter a downward trend was observed [26]. Age-dependent declines in SEB-induced IL-10 and FoxP3+ T regs, that may reflect non-specific regulatory responses, may be paralleled by a reciprocal increase in antigen-specific regulation (i.e. frequencies of IL-10 expressing Tr1 cells and antigen-specific production of IL-10 by T cells) reflecting an increasingly important role of antigen-specific regulation with age.

## 5 Conclusions

The present study investigated the relative impact of age on the development of immune responses during early childhood in urban and rural populations in the Tropics. Our data provide evidence of continuing development of immunity up to 5 years of age (i.e. increasing percentages of memory CD4^+^ and CD8^+^ T cells and decreasing percentages of naïve CD4^+^ T cells) but also an age-dependent down-regulation of pro-inflammatory responses to innate immune stimuli, particularly for IL-8. These effects were paralleled by declines in some immune regulatory markers (i.e. SEB-induced IL-10 and percentages of FoxP3^+^ T regs). Environment, defined by urban versus rural, appeared to have relatively small effects on immune responses in our study population compared to age. Importantly, the data indicate the existence of a hyper-responsive innate immune response in early life that is down-regulated by age. Such a response may be important for host defense against pathogens in the absence of effective adaptive immunity but may run the risk of immunopathology (e.g. viral-induced inflammation of the respiratory tract) and, therefore, is down-regulated in parallel with the development of a more targeted and regulated adaptive immune response.

## Figures and Tables

**Table 1:** Baseline characteristics of study population.

Characteristic	Rural (*N* = 111)	Urban (*N* = 129)	*P* value
Age (months)			
Median (range)	25 (6–60)	25 (6–60)	
Sex			
Male/female (%)	49.6/40.4	37.2/62.8	0.04
Maternal educational level (%)			
Don't know	0.9	0.8	
Illiterate	4.5	3.2	
Primary complete	38.7	28.4	
Secondary complete	55.9	67.6	0.16
Monthly income (US$)			
Median (range)	100 (20–500)	100 (20–1500)	0.10
Breastfeeding duration (months)			
Don't know	1.8%	0%	
< 6 months	3.7%	5.6%	
6–12 months	63.3%	68.2%	
13–24 months	29.4%	25.4%	
> 24 months	1.8%	0.8%	0.43
Crowding (persons/sleeping room)			
Median (range)	2.7 (0.7–9.0)	3.0 (0.8–12.0)	
Birth order			
1st–2nd	43.3%	51.2%	
3rd–4th	27.0%	25.2%	
≥ 5th	29.7%	23.6%	0.43
Bathroom (%)			
Field	27.9%	4.7%	
Latrine	72.1%	37.8%	
WC	0%	57.5%	< 0.001
Drinking water, sources^a^			
Rain (%)	85.6%	1.6%	< 0.0001
River (%)	93.7%	0%	< 0.0001
Well (%)	26.1%	0%	< 0.0001
Piped (%)	36.9%	17.1%	0.001
Potable (%)	0%	81.4%	< 0.0001
House construction materials (%)			
Wood/bamboo	43.2	33.1	
Wood/cement	41.5	33.9	
Cement/brick	14.4	31.5	
Other	0.9	1.5	0.02
Electrical appliances, number			
0	32.4	27.1	
1–2	28.8	22.5	
3–4	38.8	50.4	0.2

a Households, especially in the rural area, have multiple sources of drinking water that vary between wet and dry seasons.

**Table 2:** Nutritional, hematologic, and allergic characteristics by age and residence groups.

Study group	Rural			Urban		
	6–9 months (*N* = 29)	22–26 months (*N* = 37)	48–60 months (*N* = 45)	6–9 months (*N* = 34)	22–26 months (*N* = 43)	48–60 months (*N* = 52)
Age						
Median	7	24	56	8	25	53
Weight (kg)						
Median (range)	7.8 (6.0–9.4)	12.0 (8.3–14.6)	16.8 (13.3–43.1)	7.5 (5.7–9.6)	11.3 (8.6–14.5)	15.5 (12.8–19.3)
Hematocrit (%)						
Median (range)	36 (30–42)	38 (31–44)	38 (32–43)	36 (30–40)	38 (32–45)	38 (34–44)
White cell count (× 10^6^ cells/mL)						
Median (range)	7700 (4900–15200)	7600 (4600–14700)	6900 (5000–18300)	7350 (5000–13200)	7100 (4000–14100)	7000 (4800–12000)
Lymphocyte count (× 10^6^ cells/mL)						
Median (range)	5126 (864–8873)	4690 (1610–8512)	3762 (2000–6489)	5522 (2856–11220)	3740 (2160–6345)	3286 (1120–5928)
Neutrophil count (× 10^6^ cells/mL)						
Median (range)	2156 (588–8512)	2392 (690–6786)	2871 (672–12078)	1739 (584–4617)	3108 (1120–6100)	3483 (637–8512)
Eosinophil count (× 10^6^ cells/mL)						
Median (range)	62 (0–304)	156 (0–1496)	121 (0–1700)	63 (0–396)	100 (0–2961)	129 (0–714)
Geohelminths (%)	a2		a2	a2	a6	a1
Any infection	3.6%	62.2%	53.5%	9.4%	35.1%	43.1%
*A. lumbricoides*	0%	37.8%	20.9%	0%	22.9%	22.5%
*T. Trichiura*	0%	59.5%	53.5%	3.6%	20.0%	36.7%
*S. stercoralis*	0%	0%	2.3%	0%	0%	0%
SPT (%)	a *8	a *5	a *13	a *4	a *8	a *9
Any allergen	0%	3.1%	9.4%	3.3%	11.4%	10.4%
House dust mite	0%	3.1%	9.4%	0%	11.4%	8.3%
Others	0%	0%	6.3%	3.3%	0%	2.1%
Wheeze, past 12 months (%)	34.5%	29.7%	17.8%	29.4%	45.2%	17.6%

SPT—allergen skin prick test reactivity, Wheeze, past 12 months—episode of wheeze within past 12 months.

a Missing values.

**Table 3:** Cytokine (IL-6, IL-10, and TNF-α) and chemokine (IL-8) responses to TLR agonists. Shown are median values (interquartile range). Bold italics represent significant differences (at *P* < 0.01) between urban and rural infants aged 6–9 months. Numbers in bold (no italics) represent significant differences (at *P* < 0.01) across the 3 age groups; 6–9 (All), 22–26, and 48–60 months.

Immune parameter for innate responses		6–9 months (median [IQR])			22–26 months (median [IQR])	48–60 months (median [IQR])
		Urban (*N* = 34)	Rural (*N* = 29)	All (*N* = 63)	All (*N* = 80)	All (*N* = 97)
TLR1/2						
IL-6	ng/ml	6.5	8.4	7.2	4.8	5.3
		[5.8–8.6]	[7.6–9.6]	(6.1–9.6)	[3.3–9.6]	[3.5–7.3]
IL-8	ng/ml	***46.5***	***87.8***	**64.6**	**35.3**	**27.2**
		[39.6–64.8]	[73.8–97.1]	[43.4–91.8]	[17.9–56.8]	[20.2–45.3]
TNF-α	pg/ml	519	1,233	671	428	374
		[340–789]	[671–2500]	[340–1439]	[217–1037]	[221–914]
IL-10	pg/ml	399	431	**419**	**237**	**213**
		[312–550]	[401–480]	[317–550]	[125–406]	[171–387]
TLR3						
IL-6	pg/ml	259	517	358	276	269
		[77–503]	[151–955]	[77–68]	[0–681]	[34–661]
IL-8	pg/ml	***255***	***3,399***	572	329	177
		[67–572]	[1885–6503]	[112–2255]	[46–684]	[48–555]
TNF-α	pg/ml	64	418	205	176	45
		[7–326]	[79–865]	[27–461]	[6–347]	[0–213]
IL-10	pg/ml	0	0	0	0	0
		[0–0]	[0–1]	[0–0]	[0–0]	[0–0]
TLR4						
IL-6	ng/ml	28.4	26.7	**28.1**	**21.8**	**19.2**
		[22.0–37.6]	[14.9–18.3]	[22.0–37.6]	[15.9–30.4]	[15.3–28.2]
IL-8	ng/ml	***51.7***	***87.4***	**66.6**	**42.4**	**34.9**
		[37.0–71.2]	[60.1–98.7]	[40.7–82.3]	[21.2–65.8]	[20.9–54.1]
TNF-α	ng/ml	7.6	9.9	8.3	7.0	6.2
		[3.9–10.0]	[9.7–10.0]	[5.7–10.0]	[3.9–10.0]	[4.1–9.9]
IL-10	ng/ml	2.0	1.9	**2.0**	**1.8**	**1.4**
		[1.6–2.0]	[1.8–2.0]	[1.6–2.0]	[1.1–2.0]	[1.1–1.9]
TLR5						
IL-6	ng/ml	18.4	16.2	17.9	15.2	13.6
		[16.9–23.20	[14.9–18.3]	[15.2–23.2]	[7.7–19.6]	[8.8–18.7]
IL-8	g/ml	***69.5***	***92.9***	**77.9**	**42.6**	**37.1**
		[48.9–77.5]	[87.4–98.7]	[63.7–100.0]	[32.4–75.1]	[17.0–55.4]
TNF-α	ng/ml	3.3	3.2	3.6	2.3	2.6
		[2.2–5.2]	[2.0–6.2]	[2.2–6.2]	[1.2–4.8]	[1.3–4.9]
IL-10	ng/ml	1.5	1.7	**1.6**	**1.0**	**1.1**
		[1.1–1.9]	[0.9–2.0]	[0.9–2.0]	[0.6–1.6]	[0.6–1.4]
TLR6						
IL-6	ng/ml	1.8	1.9	**1.8**	**0.8**	**0.7**
		[1.0–2.3]	[1.3–3.0]	[1.0–2.6]	[0.4–2.3]	[0.2–1.1]
IL-8	pg/ml	18.7	31.2	**27.8**	**9.8**	**5.0**
		[12.0–33.0]	[27.7–55.6]	[15.6–38.9]	[3.8–21.8]	[2.5–10.7]
TNF-α	pg/ml	57	121	**79**	**11**	**0**
		[3–250]	[73–682]	[28–294]	[0–5]	[0–45]
IL-10	pg/ml	88	102	**100**	**41**	**14**
		[14–222]	[74–145]	[37–174]	[0–107]	[0–57]
TLR9						
IL-6	pg/ml	0	0	0	0	0
		[0–12]	[0–26]	[0–0]	[0–0]	[0–0]
IL-8	pg/ml	777	763	**777**	**351**	**78**
		[334–1637]	[671–2,500]	[241–1,374]	[96–607]	[25–39]
TNF-α	pg/ml	0	0	0	0	0
		[0–0]	[0–18]	[0–0]	[0–0]	[0–0]
IL-10	pg/ml	0	0	**0**	**0**	**0**
		[0–16]	[0–31]	[0–28]	[0–0]	[0–0]

**Table 4:** Functional markers of adaptive immunity shown by cytokine responses by SEB-stimulated peripheral blood leukocytes. Shown are median values (inter-quartile range). Bold italics represent significant differences (at *P* < 0.01) between urban and rural infants aged 6–9 months. Numbers in bold (no italics) represent significant differences (at *P* < 0.01) across the 3 age groups; 6–9 (All), 22–26, and 48–60 months.

Immune parameter	6–9 months (median [IQR])			22–26 months (median [IQR])	48–60 months (median [IQR])
	Urban (*N* = 34)	Rural (*N* = 29)	All (*N* = 63)	All (*N* = 80)	All (*N* = 97)
SEB-induced cytokines (pg/ml)					
IFN-γ	***24,349***	***13,259***	21,204	21,589	19,709
	[16,789–34,159]	[7759–23,294]	[11,309–28,369]	[14,569–38,619]	[12,239–32,829]
IL-5	1,472	1,291	**1,354**	**3,305**	**3,205**
	[908–2350]	[767–1874]	[828–2098]	[2040–4463]	[2077–4416]
IL-13	4677	5398	4834	5647	5530
	[3623–6626]	[4122–6415]	[3676–6626]	[3567–4812]	[3442–7597]
IL-10	***1,509***	***576***	**1,341**	**886**	**406**
	[811–2546]	[45–1616]	[286–2083]	[223–2141]	[41–1186]

**Table 5:** Phenotypic markers of adaptive immunity. Shown are median values (inter-quartile range). Bold italics represent significant differences (at *P* < 0.01) between urban and rural infants aged 6–9 months. Numbers in bold (no italics) represent significant differences (at *P* < 0.01) across the 3 age groups; 6–9 (All), 22–26, and 48–60 months.

Immune parameter	6–9 months (median [IQR])			22–26 months (median [IQR])	48–60 months (median [IQR])
	Urban (*N* = 34)	Rural (*N* = 29)	All (*N* = 63)	All (*N* = 80)	All (*N* = 97)
CD4+					
Naïve (CD4^+^CD45RA^+^CD28^+^)					
Percentages (%)	71.1 [60.4–73.9]	72.8 [67.8–76.0]	**71.5** [65.3–75.6]	**60.5** [47.1–68.7]	**53.3** [46.0–61.2]
Cell counts (× 106 cells/mL)	1543 [996–1897]	1425 [952–1964]	1474 [991–1943]	950 [515–1227]	670 [403–877]
Memory (CD4^+^CD45RA^−^CD28^+^)					
Percentages (%)	17.6 [14.5–22.0]	18.2 [15.0–20.0]	**17.9** [14.6–21.4]	**25.1** [21.0–29.8]	**35.1** [29.3–40.3]
Cell counts (× 10^6^ cells/mL)	355 [259–501]	360 [252–448]	**355** [252–478]	**385** [303–532]	**392** [301–521]
CD4^+^CD45RA^+^CD28- #					
Percentages (%)	9.4 [6.1–13.1]	7.1 [4.9–9.8]	8.3 [5.2–11.3]	8.6 [5.7–14.1]	8.7 [5.2–12.4]
Cell counts (× 10^6^ cells/mL)	207 [102–325]	123 [101–178]	**150** [101–257]	**135** [73–273]	**94** [59–145]
Regulatory (CD4^+^CD25^+^FoxP3^+^)					
Percentages (%)	4.1 [2.6–4.8]	3.9 [3.3–5.4]	**4.0** [2.8–5.0]	**2.7** [1.8–4.0]	**2.6** [2.1–3.3]
Cell counts (× 10^6^ cells/mL)	67 [43–95]	58 [49–103]	**63** [47–100]	**33** [23–53]	**25** [16–39]

CD8^+^					
Naïve (CD8^+^CD45RA^+^CD28^+^)					
Percentages (%)	37.3 [17.1–49.4]	35.0 [25.1–52.3]	35.9 [26.9–49.5]	29.7 [21.5–44.4]	31.0 [21.8–42.0]
Cell counts (× 10^6^ cells/mL)	397 [213–560]	380 [223–526]	389 [216–533]	299 [193–404]	284 [175–380]
Memory (CD8^+^CD45RA^−^CD28^+^)					
Percentages (%)	***3.9*** [2.7–6.9]	***6.5*** [4.7–10.1]	**5.1** [3.4–8.3]	**7.9** [5.6–10.1]	**9.2** [7.0–12.5]
Cell counts (× 10^6^ cells/mL)	49 [25–68]	65 [42–123]	**55** [30–80]	**75** [43–112]	**86** [50–118]
CD8^+^CD45RA^+^CD28^−^a					
Percentages (%)	57.4 [46.7–73.8]	60.8 [36.8–71.8]	59.2 [43.9–72.7]	59.1 [42.1–74.3]	59.8 [45.0–70.2]
Cell counts (× 10^6^ cells/mL)	577 [378–879]	678 [238–931]	**609** [370–905]	**513** [344–815]	**447** [288–654]

a This population may represent effector cells [10,11].

**Table 6:** Associations between environmental exposures and SEB-induced IL-10. Model 1 was adjusted for age, sex, and maternal educational level. Model 2 was adjusted for age, sex, maternal educational level and other covariates excluding residence. Shown are Fold change (FC) and 95% confidence intervals (95% CI). Environmental exposures with significant effects (*P* < 0.01) are shown in bold. Univar.—univariate analyses; Primary—completed primary education.

Exposure/factor	Analysis	SEB-induced IL-10		
		FC	95% CI	*P* value
Age	Univar.	**0.50**	**0.33–0.76**	**0.001**
Sex	Univar.	1.83	0.02**–**3.66	0.09
Maternal education (Illiterate vs. primary)	Univar.	0.86	0.36**–**2.10	0.75
Residence (urban vs. rural)	Univar.	**8.56**	**4.61–15.91**	**< 0.001**
Crowding	Model 1	**1.26**	**1.06–1.51**	**0.01**
	Model 2	**1.35**	**1.12–1.62**	**0.002**
Piped water	Model 1	**0.32**	**0.15–0.68**	**0.003**
(yes vs. no)	Model 2	0.37	0.17**–**0.84	0.02
Birth order	Model 1	1.08	0.55**–**2.13	0.83
(≥ 3 rd vs. 1st/2nd)	Model 2	0.83	0.39**–**1.73	0.61
Breast-feeding	Model 1	0.80	0.35**–**1.74	0.54
(≥ 13 months vs. ≤ 12 months)	Model 2	0.70	0.31**–**1.57	0.39
Bathroom	Model 1	0.41	0.16**–**1.06	0.07
(field vs. other)	Model 2	0.52	0.19**–**1.41	0.20
House construction	Model 1	1.05	0.47**–**2.33	0.91
(cement vs. wood)	Model 2	0.21	0.52**–**2.82	0.65
Geohelminth infection	Model 1	0.50	0.23**–**1.08	0.08
(yes vs. no)	Model 2	0.49	0.24**–**1.07	0.07
